# Progress towards malaria elimination in Sabang Municipality, Aceh, Indonesia

**DOI:** 10.1186/1475-2875-12-42

**Published:** 2013-01-30

**Authors:** Herdiana Herdiana, Anis Fuad, Puji BS Asih, Siti Zubaedah, Risalia Reni Arisanti, Din Syafruddin, Hari Kusnanto, Maria Endang Sumiwi, Titik Yuniarti, Ali Imran, Rahmadyani Rahmadyani, Muhammad Yani, Rita Kusriastuti, Siti Nadia Tarmizi, Ferdinand J Laihad, William A Hawley

**Affiliations:** 1 Child Survival and Development Cluster, UNICEF Indonesia Country Office, Jalan Sudirman Kav. 31, Wisma Metropolitan II, Fl 10th, Jakarta, 12920, Indonesia; 2 Faculty of Medicine, University of Gadjah Mada, Yogyakarta, Indonesia; 3 Eijkman Institute for Molecular Biology, Jalan Diponegoro, 69, Jakarta, 10430, Indonesia; 4 Ministry of Health, Directorate of Vector Borne and Zoonotic Diseases, Jakarta, Indonesia; 5 Municipal Health Authority of Sabang, Jalan By Pass Cot Ba’U, Sabang, Province of Aceh, Indonesia; 6 Provincial Health Authority of Aceh Province, Banda Aceh, Aceh Province, Indonesia

**Keywords:** Malaria control, Malaria elimination, Sabang municipality, Aceh, Indonesia

## Abstract

**Background:**

Indonesia has set 2030 as its deadline for elimination of malaria transmission in the archipelago, with regional deadlines established according to present levels of malaria endemicity and strength of health infrastructure. The Municipality of Sabang which historically had one of the highest levels of malaria in Aceh province aims to achieve elimination by the end of 2013.

**Method:**

From 2008 to 2010, baseline surveys of malaria interventions, mapping of all confirmed malaria cases, categorization of residual foci of malaria transmission and vector surveys were conducted in Sabang, Aceh, a pilot district for malaria elimination in Indonesia. To inform future elimination efforts, mass screening from the focal areas to measure prevalence of malaria with both microscopy and PCR was conducted. G6PD deficiency prevalence was also measured.

**Result:**

Despite its small size, a diverse mixture of potential malaria vectors were documented in Sabang, including *Anopheles sundaicus*, *Anopheles minimus*, *Anopheles aconitus* and *Anopheles dirus*. Over a two-year span, the number of sub-villages with ongoing malaria transmission reduced from 61 to 43. Coverage of malaria diagnosis and treatment, IRS, and LLINs was over 80%. Screening of 16,229 residents detected 19 positive people, for a point prevalence of 0.12%. Of the 19 positive cases, three symptomatic infections and five asymptomatic infections were detected with microscopy and 11 asymptomatic infections were detected with PCR. Of the 19 cases, seven were infected with *Plasmodium falciparum*, 11 were infected with *Plasmodium vivax*, and one subject was infected with both species. Analysis of the 937 blood samples for G6PD deficiency revealed two subjects (0.2%) with deficient G6PD.

**Discussion:**

The interventions carried out by the government of Sabang have dramatically reduced the burden of malaria over the past seven years. The first phase, carried out between 2005 and 2007, included improved malaria diagnosis, introduction of ACT for treatment, and scale-up of coverage of IRS and LLINs. The second phase, from 2008 to 2010, initiated to eliminate the persistent residual transmission of malaria, consisted of development of a malaria database to ensure rapid case reporting and investigation, stratification of malaria foci to guide interventions, and active case detection to hunt symptomatic and asymptomatic malaria carriers.

## Background

Malaria is a major public health problem in most tropical countries, including Indonesia. Recent models estimate that over 105 million of Indonesia’s 239 million population are at risk for malaria infection, with transmission varying widely across this most populous entirely tropical country [1]. Though formally reported deaths are less than 1,000 annually [2], recent work estimates that about 11,000 people die annually due to infections with *Plasmodium falciparum *[3]. The number of deaths caused by *Plasmodium vivax* infection is not known. The most recent WHO estimate of the number of deaths due to malaria is approximately 3,000 per year in Indonesia [2]. In 2009, the Indonesian MOH explicitly made malaria elimination a national goal, to be accomplished in a step-by-step, island-by-island fashion over the next several decades [4].

Sabang municipality in Aceh Province was once one of the most malarious areas in Indonesia [5]. Now, after successful control, with only sporadic focal transmission occurring, Sabang’s government [6], with support from provincial and central authorities, is committed to eliminate malaria transmission by the end of the year 2013 [7].

This effort is in line with the recent rise of malaria elimination in the global public health agenda, signaled by the commitment of the 60th World Health Assembly in 2007 that all countries should commit to eliminate malaria by 2050 [8]. Indonesia aims to eliminate malaria by 2030 [4], while the target for low-endemic Aceh Province is 2015 [4,7]. In 2009, the Provincial Parliament of Aceh endorsed this plan, and budgeted significant resources towards its achievement. In addition, the Governor of Aceh has provided legal support to the plan via a new regulation promulgated in 2010 [7].

If the political and legal framework for malaria elimination in Indonesia and Aceh is in place, clear technical challenges remain. Indonesia straddles Wallace’s line, and has perhaps two dozen known malaria vectors with a large variety of behaviours. Further, Indonesia has a high prevalence of both *P*. *falciparum* and *P*. *vivax*, which tremendously complicates the elimination effort [9,10]. Though decentralization of the government in 2000 (including decentralization of the health system) has resulted in some deterioration of services, some well-governed districts have risen to the challenge and have improved health service delivery [11].

In the context of Indonesia, Sabang District is an appropriate place to pilot malaria elimination by virtue of its geographic position at the western end of the archipelago, its diverse mosquito fauna, the presence of both major malaria parasites, and its strong local government. The rate of importation of malaria into Sabang is likely low, giving hope that the municipality will be able to maintain elimination in the long run. Sabang has, in general outline, followed the recommendations of the WHO for malaria elimination [8], while recognizing the necessity to adapt these guidelines according to circumstances on the ground in Sabang.

The effort has been led by the government of Sabang, with support from the Provincial Health Office of Aceh, the National Malaria Control Program of Indonesian Ministry of Health, University of Gadjah Mada, Eijkman Institute, the World Health Organization (WHO) and the United Nation Children Fund’s (UNICEF).

This account begins with a description of what WHO terms the ‘control’ phase of malaria control [8], in which post-tsunami funding allowed rapid scale-up of IRS and LLINs, improved malaria diagnosis, and treatment with ACT. The rapidly achieved high coverage ot these interventions resulted in a quick decline in malaria cases, but these persist at a low level [6]. The reorientation of activities aimed at moving the programme towards malaria elimination is then described. These efforts included intensive mapping of both vectors and malaria cases, establishment of a comprehensive database, and most recently, aggressive active case finding coupled with surveys to estimate G6PD prevalence and malaria parasite genotypes.

## Methods

### Site

**Figure 1:**
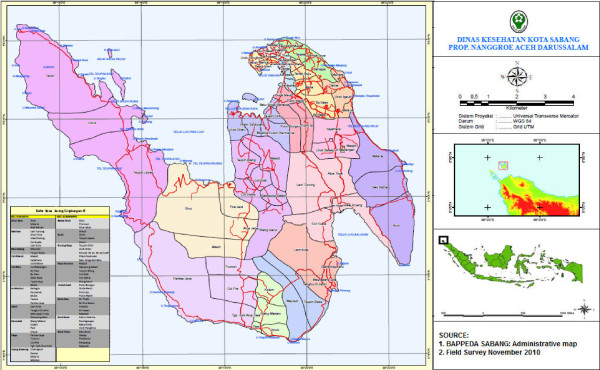
Map of Sabang Municipality and Weh Island showing position of Sabang in Indonesia and sub villages within the municipality.

The municipality of Sabang is located at the north-westernmost part of Indonesia, and is part of Aceh Province (Figure 1). The district has an area of 153 km2 covering five islands: Weh, Klah, Rubiah, Seulako, and Rondo [12]. Only the largest island, Weh, is permanently inhabited, though Rondo has a lighthouse attendant and Rubiah has inexpensive tourist shelters that are sometimes occupied. Thus, all but one of the municipality’s official residents totaling 30,653 people live on Weh Island, which is divided administratively into two subdistricts and 18 villages [13].

**Figure 2:**
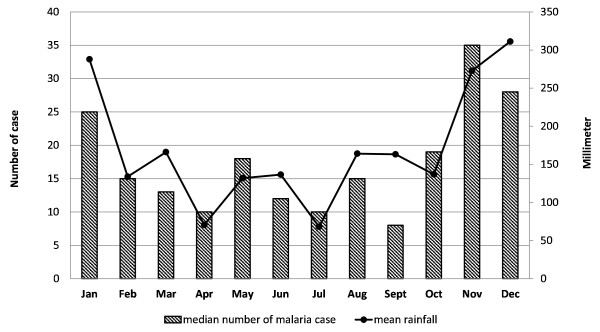
Average monthly rainfall and median monthly malaria cases in Sabang from 2005 – 2009.

Malaria occurs year-round in Sabang (Figure 2). An increase in cases is observed during the rainy season, but rain and malaria occur every month.

### History of malaria control in Sabang

Malaria in Sabang was recognized by the government of Indonesia after an outbreak was reported in 1967 [5]. In 1970, Sabang was included in phase II of the Government of Indonesia’s Malaria Eradication Programme (KOPEM), which was based on spraying with DDT (2 gr/m2 active ingredient) twice a year [14].

DDT spraying was effective, resulting in a drop in the prevalence of parasitaemia in school children from 37.0% in 1971 to 1.6% in 1975. It remained below 7.5% until 1978. During the early 1980s, the prevalence of parasitaemia in the overall population ranged from 7.5% to 10.4% [14]. After the malaria eradication campaign was abandoned, malaria remained as a major public health problem in Sabang. The situation worsened with appearance of choloroquine-resistant and sulphadoxine-pyrimethamine-resistant in *P. falsiparum* Sabang in the late 1980s [15].

From 1990 to 2004, the malaria control programme in Sabang still emphasized IRS, though DDT was no longer used after 1994 [16]. From 1995 to 2000, Bendiocarb® was widely used in Sabang, while Vectron® (etofenprox) was used from 2001 to 2005. Despite the documented resistance of *P*. *falciparum* to chloroquine and SP, these drugs were still used for malaria treatment [17].

**Figure 3:**
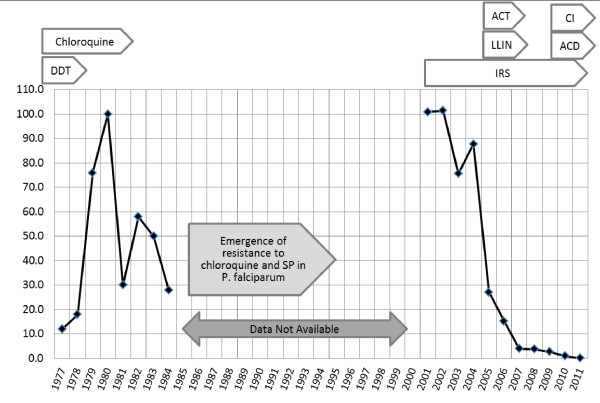
**Confirmed malaria cases per thousand population in Sabang based upon available passive surveillance data, showing major interventions and drugs used for treatment from 1977 to 2011.** DDT: dichlorodiphenyltrichloroethane, ACT: Artemisinin-based Combination Therapy, LLIN: Long Lasting Insectiside Treated Bednets, IRS: Indoor Residual Spraying, CI: Case Investigation, ACD: Active Case Detection

On 26 December 2004, the Indian Ocean tsunami devastated much of the west coast of Aceh, including parts of Sabang. Though the health infrastructure of the province was largely destroyed, with loss of both material and personnel, aid from the central government of Indonesia, various UN agencies and NGOs supported rapid recovery of the malaria programme and improvements in health infrastructure. Improvements in malaria control included an extensive IRS programme in the year immediately following the tsunami, large scale LLIN distribution, and a change in malaria treatment policy to ACT as first-line treatment for uncomplicated malaria. These efforts resulted in a rapid decrease in malaria, with reported incidence declining from 87.8 infections per 1,000 population in 2004 to four cases per thousand in 2007. The proportion of infections due to *P*. *falciparum* declined from 77% to 64% from 2002 to 2007. No fatalities due to malaria have occurred post-tsunami [17]. The confirmed cases and interventions are depicted in Figure 3.

The studies of of Swellengrebel and Rodenwaltd in 1932 indicated four *Anopheles* species on Sabang, *Anopheles minimus*, *Anopheles sundaicus*, *Anopheles subpictus* and *Anopheles vagu*s, while Hudson *et al*. identified five *Anopheles* species on Sabang during a dry season survey in 1985: *Anopheles aconitus*, *Anopheles leucophyrus*, *Anopheles maculatus*, *Anopheles minimus* and *Anopheles sundaicus*. Hudson *et al*. came to no definite conclusion as to the primary vectors due to small numbers of adults captured [14]. Since Hudson’s work, no published vector evaluation report exists in local government archives.

The elimination programme was launched by the Indonesian Ministry of Health in 2008, and was followed by local government in 2009 [17]. With firm commitment and support from national, provincial, and district authorities, the District Health Office of Sabang was tasked with designing an effective technical approach that would lead the district to malaria elimination. After consultation with national malaria control programme, WHO experts, and UNICEF, four initial activities were planned and executed; e.g. mapping of malaria vectors; mapping of malaria cases; determination of residual foci of malaria transmission; and mass population screening to detect asymptomatic cases. These activities, which were carried out from 2008 to 2010, are described below.

### Vector assessment

Mosquito surveys were done in all villages in Sabang from August – November 2008. Surveys were carried out in each of 18 villages.

All night human landing collections were carried out in three houses in each village, with one person indoors and one person outdoors in each of the three houses. Collections were carried out from 7:00 pm until 7:00 am over a period of 18 nights. As sunset in Sabang is approximately 6:45 pm and sunrise is at 6:30 am, collections were carried out from just after dusk until just after dawn. Collectors used aspirators to catch mosquitoes coming to bite. Mosquitoes were stored in labeled cups for further processing.

During night collections, collectors worked for 10 minutes each hour collecting indoor resting mosquitoes using an aspirator. Morning resting collections were carried out indoors and outdoors near the night collection stations.

Larval collections aimed to sample as great a variety of habitats as possible with particular attention to areas known to have ongoing malaria transmission. *Anopheles* larvae were counted and taken to the laboratory for identification. Larval habitats were characterized for the following: type of sites; distance to the nearest house, water quality (clear, cloudy or muddy), amount of sunlight (full sun, full shade or mixed), presence and type of vegetation, and the presence of other mosquito genera and invertebrate predators [18,19]. GPS coordinates were recorded for each site sampled.

All adult mosquitos were morphologically identified in the laboratory using keys developed by Reid [20] and O’Connor & Soetanto [21]. Hourly man biting rates indoors and outdoors were calculated; host preferences; and indoor and outdoor resting habits were described [22].

### Development of malaria map and database

To develop the basis for surveillance and rapidly respond to infections, available malaria baseline data at household level were incorporated into a GIS system to support implementation of malaria elimination. Design of the database included wide consultation, including focus group discussions with the District Planning Board, Civil Registration Office, District Health Office, Health Division of the Armed Forces, Center of Data and Information under the Mayor of Sabang, and sub district leaders. Both routine and survey data were incorporated into the database. A cross sectional census and survey in six villages selected on the basis of their continued malaria endemicity over the most recent three-year period was conducted. The survey was carried out from March to June 2009 in the villages of Paya Seunara, Balohan, Jaboi, Batee Shok, Krueng Raya and Keneukai. The questionnaire included demographic data, malaria risk factors and measurement of coverage of IRS and LLINs. All houses in each village were surveyed and mapped with GPS.

In addition to the household survey, routine surveillance data from the municipal health authority from health centres for 2007 and 2008 were reviewed, collated and incorporated into the database. Malaria cases were listed and each household visited. A standard questionnaire was administered to the index case or an older relative in instances where the case was a minor. Information from the interview and GPS coordinates were entered into the database [23]. Information on population characteristics from civil authorities of Sabang was also incorporated in the database.

A desk review of secondary data from 2009 and 2010 to determine foci of malaria transmission was conducted. Foci was stratified and mapped twice, in July 2009 and December 2010 [17].

Analysis was carried out and maps and graphics produced using spatial analysis software (SatScan), Epi Info version 3.3.2 and Excel.

### Mass blood screening

In May to October 2010, mass blood screening in 14 villages with known malaria transmission was carried out. Urban areas with no recorded transmission were excluded from the survey. In all, 14,462 individuals were surveyed from the target population of 19,443 (74% of target). For these samples, the village of origin of each subject was known. An additional 1,767 subjects in schools and government offices for which unrecorded household of residence were also surveyed. For each individual, thick and thin blood smears and blood spots with filter paper No.1 (Whatman International, Maidstone, Kent, UK) were collected [24]. All slides were reviewed by expert microscopists. Location of confirmed positive cases was geocoded with GPS. All positive cases were confirmed with PCR. 1,181 negative samples from asymptomatic subjects were tested with PCR, with the samples selected weighted towards villages with known positive cases [25].

Blood samples from randomly-selected household in this survey were checked for the presence of glucose-6-phosphate dehydrogenase (G6PD) deficiency. For this test, 5 μl of whole blood was taken by finger puncture with a micropipette and a disposable tip, and mixed with the reaction solution for G6PD in a 1.5-ml microcentrifuge tube (Eppendrof). In the field, qualitative assays used water-soluble tetrazolium salt, WST-8, that also produces water-soluble formazan which are included in the Dojindo test kit (Dojindo Laboratories, Tokyo, Japan) [26].

Additionally, all dried blood spots in filter paper were sent to the Eijkman Institute, Jakarta for molecular analysis. DNA was extracted by chelex-100 ion exchanger (Biorad Laboratories, Hercules, CA) [27,28]. Three exons were analysed using polymerase chain reaction amplification and restriction fragment length polymorphisms (PCR/RFLP) to determine the most common known mutations for G6PD deficiency in Indonesia such as (Mediterranian (563C/T), Coimbra (592 C/T), Vianchang Canton (1376 G/T), Vanua Lava (383 T/C), Mahidol (487 G/T), Chatam (1003 G/A), Kaiping (1388 G/A), Vianchang (871 G/A, 1311 C/T) [29-31].

## Results

### Vector assessment

Twenty-three adult *Anopheles* were collected during six nights of all night collections, including 11 *An*. *aconitus*, four *Anopheles dirus*, three *Anopheles flavirostris*, two *An*. *sundaicus*, one each of *An*. *subpictus* and *An*. *minimus* and one unidentified. Four anophelines were captured indoors (*An*. *subpictus*, *An*. *flavirostris* and *An*. *aconitus*) and 19 were collected outdoors (*An*. *dirus*, *An*. *sundaicus*, *An*. *flavirostris*, *An*. *minimus*, *An*. *aconitus*). All mosquitoes were collected after 9 pm. No mosquitoes were collected in the resting collections. Adult *Anopheles* mosquitoes were captured in the villages of Krueng Raya, Anoi Itam, Jaboi, Iboih, Paya Seunara and Batee Shok. A table of breeding sites with Anophelines and villages where adult and larval Anopheline mosquitoes were captured is shown in Tables 1 and 2.

**Table 1:** Results of larval surveys in Sabang

		**Larva surveys**		
**Type sites inspected**	**No. sites inspected**	**No. sites with Anopheles**	**%**	**Type species**
Stream	43	12	28%	An. aconitus; An. barbirostris; An. montanus; An. subpictus; An. umbrosus; An. vagus
Ditch	28	5	18%	An. vagus
Pond	41	12	29%	An. barbirostris; An. montanus; An. subpictus; An. sundaicus; An. vagus
Ground pool	32	17	53%	An. barbirostris; An. sundaicus; An. umbrosus; An. vagus
Swamp	2	1	50%	An. dirus;
Water pool	23	5	22%	An. aconitus: An. kochi; An. montanus; An. sundaicus; An. vagus
Lake	2	1	50%	An. barbirostris; An. vagus
Hoof-print	24	6	25%	An. montanus; An. vagus
Flower Vase	1	0	0%	-
Drum	9	0	0%	-
Seepage	2	1	50%	Anopheles Sp L2
Water container made of cement	3	0	0%	-
Lagoon	1	1	100%	An. sundaicus
**Total**	**211**	**61**		

**Table 2:** Villages with larval habitats with anophelines and results of landing and resting collection by village

**Village**	**Total no. dips**	**Larval survey**		**Type species**	**Landing catch**		**Resting**
		**No. sites inspected**	**No. sites with Anopheles**		**No. Anopheles indoors**	**No. Anopheles outdoors**	
Kota Atas	0	4	0	-	0	0	0
Kota Bawah Barat	25	5	0	-	0	0	0
Kota Bawah Timur	128	16	2	An.sundaicus	0	0	0
Aneuk Laot	91	14	3	An. dirus, An. barbirostris, An.sp	0	0	0
Krueng Raya	155	18	9	An. subpictus; An. montanus; An. aconitus; An. vagus; An.sundaicus	2	4	0
					An. subpictus (1)	An.dirus (1)	
					An.aconitus (1)	An.sundaicus (2)	
						An.flavirostris (1)	
Paya Seunara	105	11	4	An. barbirostris; An. vagus; Hyrcanus group	0	1	0
						An.flavirostris (1)	
Keunekei	75	8	7	An. umbrosus, An. sundaicus, An. vagus	0	0	0
Paya Keunekei	85	7	3	An. sundaicus; An. vagus; An. barbirostris	0	0	0
Iboih	105	13	2	An. vagus; An.sp	0	3	0
						An.dirus (3)	
Batee Shok	117	14	3	An. vagus	0	2	0
						An.minimus (1)	
						An.sp (1)	
Jaboi	111	13	6	An. aconitus; An. umbrosus; An. kochi; An. barbirostris; An. vagus; An. montanus	1	7	0
					An.aconitus (1)	An.aconitus (7)	
Cot Bau	67	11	2	An. vagus; An. sp	0	0	0
Cot Abeuk	105	11	2	An. barbirostris	0	0	0
Anoi Item	168	19	7	An. vagus; An. subpictus	1	2	0
					An.flavirostris (1)	An.aconitus (2)	
Ujung Kareng	71	13	2	An. vagus, An.sp	0	0	0
Ie Meulee	49	7	1	An. vagus	0	0	0
Beurawang	35	8	0	-	0	0	0
Balohan	114	19	8	An. vagus, An. subpictus, An.sp	0	0	0
**Total**	**1606**	**211**	**61**		**4**	**19**	**0**

**Figure 4:**
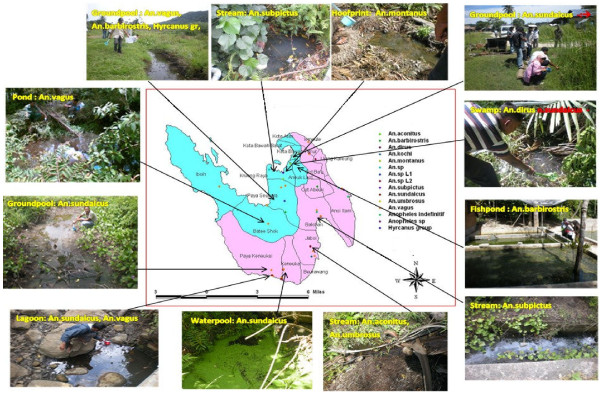
Examples of anopheles larval habitats in Sabang.

Survey teams sampled 211 potential larval habits throughout Sabang; immature stages of Anopheles mosquitoes were present in 61 (29%) sites. A total of 423 larvae and pupae were collected from 10 species: *An*. *aconitus*, *An*. *dirus*, *An*. *sundaicus*, *An*. *subpictus*, *Anopheles montanus*, *Anopheles barbirostris*, *Anopheles kochi*, *Anopheles umbrosus*, *Anopheles hyrcanus* sp, and *An*. *vagus*. This represents the first report of *An*. *dirus* from Indonesia. Details on the sites sampled are shown in Tables 1 and 2, and examples of anopheles larval habitats are depicted in Figure 4.

Paya Seunara and Krueng Raya villages were those with the greatest number of larval specimens, with 59 and 56 immatures captured, respectively [22].

### Development of malaria map and database

For the baseline malaria survey, 1,446 households from two subdistricts were interviewed and mapped using GPS. Data on demographics, malaria risk factors, and coverage of malaria prevention activities were collected using a standard questionnaire. Forty-two percent of HHs from Sukajaya Subdistrict (from the villages of Balohan, Jaboi, and Keunekei) and 58% of HHs from Sukakarya Subdistrict (from the villages of Batee Shok, Paya Seunara and Krueng Raya) were included in the survey.

Nearly all (99.5%) of respondents lived in houses with the balance living in dormitories or boarding schools. Over half of households (57%) lived in hilly areas, while 20% lived in coastal areas. Forty seven percent lived near plantations, while others lived near brushwood (16%); forest (14%); lagoon (7%); river (5%); ponds (4%); lake (0.1%) or unclassified (7%).

Most households stated that their preferred method for prevention of malaria was use of LLINs (73%), while 12% preferred use of repellents or mosquito coils, and 11% preferred elimination of larval mosquito habitats.

**Figure 5:**
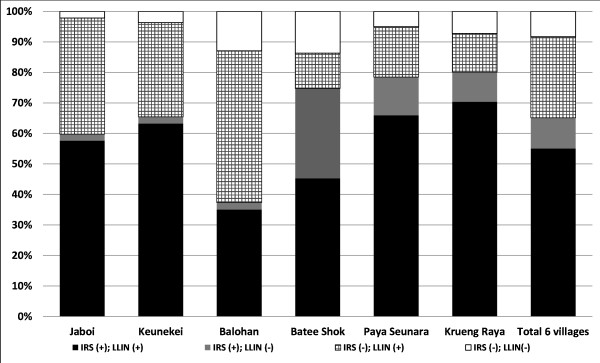
**Coverage of LLINs and IRS per household in Sabang, 2009.** Black: both interventions present; white: neither intervention present; hatched: LLINs but no IRS; gray, IRS but no LLINs.

Survey data are consistent with high coverage of both LLINs and IRS in Sabang, as summarized in Figure 5. For the six villages in the area of residual transmission in Sabang, two villages (Paya Seunara and Kreung Raya) have coverage of both IRS and LLINs over 75%, while all other villages have coverage of at least one of the two major prevention activities of over 75%. However, it was noted that 8% of houses have received neither intervention.

**Figure 6:**
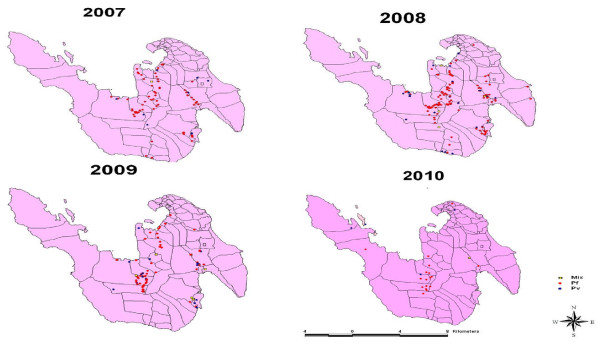
Distribution of malaria cases in Sabang from 2007 to 2010 based upon passive and active surveillance.

Malaria cases in 2007 and 2008 were mapped. In 2007, 144 cases of malaria were reported by the Municipal Health Authority (MHA) of Sabang; of these 130 were traced back to the case’s house. In 2008, 130 cases were officially reported by the MHA, but our survey team found 211 respondents who claimed that they had been diagnosed with malaria. This discrepancy is reasonable since the official data mostly depends on passively-collected primary health centre reports. In all, 319 malaria cases were geocoded, as shown in Figure 6. More than half of villages in Sabang had malaria cases, with more than half of cases in Batee Shok, Balohan, Paya Seunara, Krueng Raya, and Jaboi.

Based upon geocoded cases and population distribution, 88% of households in Sabang live within 250 metres of a malaria case, while 96% of household live within 500 metres of a malaria case. In addition, the analysis showed that 41% of households live within 250 metres of known larval habitats of Anopheles mosquitoes.

Before introduction of malaria elimination data management system, the MHA collected monthly data manually, most of which were gleaned from the microscopist’s laboratory registers. The frequency of reporting (monthly) was clearly inadequate for malaria elimination; further, no standard reporting form existed. To improve speed and accuracy of reporting of malaria cases to the MHA, a database management system was designed to allow both manual and electronic entry data at every level of the health system. The system includes information on demography, malaria cases and their therapy, laboratory data, and case follow-up. Information on epidemiologic investigations is also included, as well as survey data. The tool is a web-based application supported by MySQL database, an open source programme, which can be installed either as a standalone or a network. Output of the data can be either as raw data, graphics, maps or tables.

Though this system has been introduced to the public sector, hospitals and the private physicians remain outside the reporting system. Further, discussion showed that the MHA infrequently carried out epidemiological investigations of malaria cases in collaboration with staff from primary health care facilities. Clearly, systemic problems in coordination within the public health system remain, as do problems of communication between the public and private sectors [23].

### Stratification of foci area

**Figure 7:**
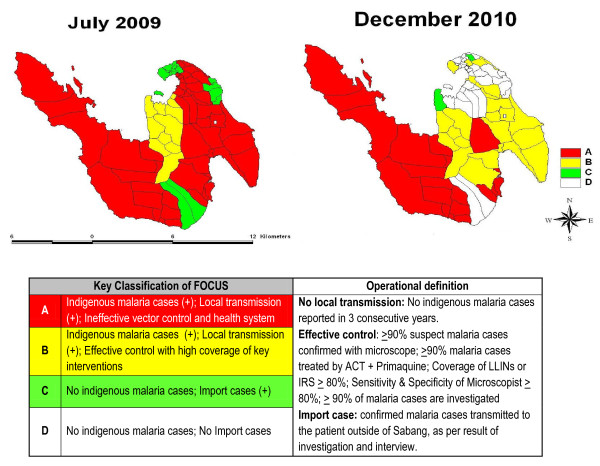
Classification of Malaria Foci in Sabang.

Stratification of malaria transmission was conducted twice by the MHA, in July 2009 and December 2011, based upon a modification of WHO criteria [32]. Definitions and distribution of sub-villages in each stratum are shown in Figure 7[7]. Of note, a reduction in the number of sub-villages in focus A and B (ongoing transmission of malaria) reduced from 61 to 43 sub-villages during the period in question.

### Mass blood screening

In 2010, 16,229 residents of Sabang were screened for malaria infection. Of these, 19 people were positive, for a point prevalence of 0.12%. Of the 19 positive cases, eight were detected via microscopy and 11 via PCR. Of 57 subjects who were feverish (temperature > 37.5 C) at the time of survey or showed a history of fever, three of 57 were positive, and these were all detected with microscopy. Of 16 asymptomatic infections, five were detected with microscopy and 11 were detected with PCR. Of the 19 cases, seven were infected with *P*. *falciparum* only, 11 were infected with *P*. *vivax* only, and one subject was infected with both species. Sixteen cases showed no symptoms or history consistent with malaria; of these 16 cases, four were infected with only *P*. *falciparum*, 11 were pure *P*. *vivax* infections and one was mixed. Fourteen of 19 infections were from one village: Batee Shok, while the remaining five positive cases were found in Iboih, Paya Kenekai, and Ie Meulee [25].

### G6PD deficiency

Subjects enrolled in the G6PD survey were the head of the household and his wife. Of 937 subjects surveyed, only two were positive (0.2%) as mild deficiency. Genomic DNA analysis revealed neither case carried the most common mutations for G6PD deficiency (Mediterranian (563C/T), Coimbra (592 C/T), Vianchang Canton (1376 G/T), Vanua Lava (383 T/C), Mahidol (487 G/T), Chatam (1003 G/A), Kaiping (1388 G/A), Vianchang (871 G/A, 1311 C/T) [25].

## Discussion

In this study, interventions were described which have dramatically reduced the burden of malaria in Sabang over the past seven years. The effect on malaria incidence has been dramatic, with a nearly 30-fold decline in incidence from 3.83 to 0.13 per thousand population from 2008 to 2011 [33]. These now-standard interventions include improved malaria diagnosis, introduction of ACT for malaria treatment, and scale-up of coverage of IRS and LLINs. The backbone of the Sabang programme has been strengthening of its diagnostic system. In 2009, twenty microscopists active in the programme were tested by an expert team according to WHO guidelines; four of the Sabang microscopists tested as expert (level 1), three at reference (level 2), eight at advanced (level 3), and two at basic (level 4). These test results show that Sabang has succeeded in training a cadre of highly proficient microscopists. By 2009, blood slides from 96% of suspected malaria cases were examined by this capable team – up from 66% in 2004. The small number of cases not confirmed via microscopy was tested using RDTs. Near universal coverage of microscopic diagnosis was coupled with introduction of use of ACT for treatment of all forms of malaria (including *P*. *vivax*) in 2005. By the end of 2009, 94% of laboratory-confirmed cases of malaria in Sabang were treated with ACT [17].

After the prohibition and cessation of DDT spraying in Indonesia in 1992, only occasional IRS was carried out in Sabang until the post-tsunami period when, from 2005 – 2007, the government of Sabang, in collaboration with the Mentor Initiative (an international NGO), carried out three-times yearly spraying with alphacypermethrin in 18 villages, with reported coverage of 50-60% [34]. LLINs were introduced to Sabang after the tsunami, with more than 15,000 nets distributed since 2005 by the government of Sabang in collaboration with CARE International and UNICEF [35]. While coverage of LLINs in the six focal villages remains high, at over 75%, data from previous surveys are mixed [23]. In July 2005, Samaritan’s Purse and Mentor reported that 98% of households owned at least one LLIN [34]. A survey by community volunteers conducted in February 2006 showed that 88% of households owned an LLIN, with reported usage rates by children under five years of age of 74% [35]. In contrast, Demographic Health Survey (DHS) results for 2007 showed that only 22% of households owned an LLIN. The likely reason for this discrepancy is that the DHS results included urban parts of Sabang, which were not targeted by the LLIN campaigns, while the previous surveys used as their sampling frame only rural areas targeted for LLIN distribution [36]. In addition to interventions targeting adult mosquitoes, focal application of the larval growth regulator pyriproxyfen was carried out in five villages from 2005 – 2007 [6].

Sabang, in accordance with official policy of the MOH of Indonesia, has taken the bold step of explicitly aiming to eliminate malaria transmission by the end of 2013 [17]. The government of Sabang is pursuing this policy aggressively, and recognizes that the key to elimination is rapid reporting and accurate information. As described above, the routine diagnosis capability with the MOH has been steadily strengthened over the past seven years. Since mid-2009, private practitioners are asked to send blood smears to government clinics for malaria diagnosis. In May 2010, the MOH set up a system of village volunteers for active case detection. One volunteer has been recruited for each subvillage. To date, 78 volunteers have been recruited, trained, and certified by the government. In the 14 villages classified as ‘active’ or ‘residual active’ (foci A and B in Figure 6) home visits are conducted twice monthly, while in non-active foci monthly visits are carried. Blood smears are taken from anyone with fever or recent history of fever and sent to the nearest community health centre. Confirmed cases are treated by health centre staff at the patient’s home, using directly observed treatment. The volunteer takes follow-up smears on days 3, 7, 14, 28 and (for P. vivax only) 90 [17].

Since August 2010, all cases of malaria detected through passive or active surveillance, via either government or private practitioners, are investigated by staff from the community health centre and the municipal health office. The investigation is adapted from the procedure used in Zanzibar [37], with four steps: 1) Interview to determine travel history, blood transfusion history, or contact with malaria patients; 2) Contact survey – taking of blood smears from family members and all neighbors within 500 meters of the index case; 3) Rapid assessment of anopheles larval habitats and biting activity by the district’s entomology team; 4) Input of information into the malaria database and classification of the case as indigenous or imported. Since 2009, every case, whether detected passively or actively, has been included in the database, with information on age, sex, occupation and other aspects that may influence a person’s malaria risk); history of disease (travel history, fever/malaria history, contact history); all information related to malaria diagnosis; treatment; notification of case and epidemiology investigation (indigenous, import, induction, introduction, relapsed), source of cases (passive case detection, active case detection, survey), location (including GPS coordinates), and nearby *Anopheles* larval habitats [17].

The emphasis on mapping larval habitats near transmission is appropriate in the context of Indonesia, as some, but not all, malaria vectors in the country are amenable to larval control via environmental management. In the malaria database, information on coverage of LLINs and IRS is linked to foci of transmission [23], so that interventions targeting larvae may be carried out should transmission continue even though coverage of interventions targeting adults is high. Effective larval control might also allow the possibility for scaling back in IRS and LLIN coverage, as continuous high coverage is difficult to support politically as malaria incidence declines to near zero levels [38].

In Sabang, as throughout Indonesia and much of southeast and south Asia, both *P*. *falciparum* and *P*. *vivax* are common [9,10,39]. The steps taken after successful control of malaria in Sabang – improved reporting, coordination with the private sector, active case detection, mapping of cases and mosquito habitats – may be sufficient to eliminate malaria on the island. However, results of our mass screening of nearly half of the population of Sabang raise doubts on the likelihood of success, especially for *P*. *vivax*. Of note, only three of the 19 cases detected in the survey were symptomatic, and these were all detected with microscopy. Of 16 asymptomatic cases, five were detected with microscopy, while the balance of 11 cases was detected with PCR. Of these 11 asymptomatic cases, 10 were pure *P*. *vivax* infections, and all of these were detected via PCR. Thus, for both species of parasite, but more so for *P*. *vivax*, subpatent, asymptomatic infections were found via PCR that were not detectable with microscopy [24]. Indeed, by extrapolation from this survey, about 165 asymptomatic infections of malaria - mostly *P*. *vivax* -- were present in Sabang at the time of the survey, all undetectable by the microscopy. Even though Sabang’s programme is based upon rapid detection, reporting, and intervention with diagnosis via conventional microscopy, it may still be possible to eliminate malaria on the island without resorting to mass treatment or expensive screening via PCR. Whether elimination is possible will depend upon the balance between the efficiency of the programme, and the rate at which subpatent, asymptomatic infections recrudesce – allowing detection and attack via medication – or spontaneously disappear [39-41]. Both processes are known to occur. An important third unknown parameter is the probability of transmission of subpatent infections to mosquitos [42]. Though presumably low, this probability can be further minimized via vector control interventions targeting both adults and larvae. These three parameters presumably vary between the two parasite species. The higher prevalence of asymptomatic *P*. *vivax* infections, coupled with the operational difficulty of radical treatment of hypnozoites, makes is likely that this parasite will be more difficult to eliminate than *P*. *falciparum*[8,39,43,44].

Aggressive efforts to treat *P*. *vivax* carry risks associated with G6PD deficiency [45,46]. Though reported prevalence in Sabang is low, based upon results of our survey, it is possible that other parts of Indonesia [29-31,47] or other parts of Asia [30,48], may have higher levels of this risk factor. Minimization of this risk will require availability of cheap and effective rapid screening tests for G6PD deficiency or better anti-hypnozoite drugs, but neither of these is available now [39,49-51]. Further, the current anti-hypnozoite drug, primaquine, must be taken over 14 days by patients that already feel recovered, raising serious problems with patient compliance [50,51].

Further complicating malaria elimination efforts in Sabang is the high mobility of Indonesians, and the fact that many Indonesians may seek employment in parts of Aceh, Kalimantan or eastern Indonesia, where risk of malaria infection is substantial. A recent imported case of malaria to Sabang in a gold miner illustrates this point. Sabang will need to establish good community based systems for screening and detection of malaria in residents returning from malaria endemic areas. Such a system has been established in Wonosobo, central Java [52], which sends many temporary workers to malaria-endemic Kalimantan. Sabang has, as yet, not set up any systems to specifically detect imported cases. Perhaps of greater importance are efforts on the part of the MOH to reduce malaria incidence throughout the country. Malaria incidence has declined in eastern Indonesia thanks to scale up of many of the same interventions rolled out in Sabang, but many pockets of high transmission remain, and these are all potential sources of imported malaria to Sabang.

## Consent

All surveillance activities described in this report were consistent with and conform to policies set by the Ministry of Health of the Republic of Indonesia.

## Abbreviations

ACT: Artemisinin-based combination therapy; DDT: Dichloro-diphenyl-trichloroethane; GPS: Global positioning system; G6PD: Glucose-6-phosphate dehydrogenase; HHs: Households; IRS: Indoor residual spraying; LLINs: Long-lasting insectide-treated nets; MHA: Municipal health authority; NGO: Non-government organization; PCR: Polymerase chain reaction; RFLP: Restriction fragment length polymorphisms; RDTs: Rapid diagnostic tests; UNICEF: United nation children’s fund; WHO: World health organization.

## Competing interest

The authors declare that they have no competing interests.

## Authors’ contributions

HH and WH wrote the whole paper. PBSA and DS contributed in mass blood screening and G6PD, HK and AF contributed in mapping malaria database, SZ, RRA, HH contributed in vector assessment. Overall survey was designed by HH, WH, RRA, MES, TY, AI, RR, MY, and FL. After survey, HH, PBSA, SZ, RRA, AF, and WH analysed, interpreted the results and drafted the manuscript. FL, RR, MY, SNT, and RK contributed in providing secondary data and inputs. All authors critically reviewed the manuscripts and approved the final version before submission.

## Funding

These studies were funded by UNICEF Indonesia.
